# Pulmonary Carcinosarcoma: A Rare Disease With Challenging Diagnosis and Treatment

**DOI:** 10.7759/cureus.26901

**Published:** 2022-07-15

**Authors:** Erinie Mekheal, Ashima Kapoor, Sherif Roman, Nader Mekheal, Christopher Millet, Marina Mekheal, Michael Maroules

**Affiliations:** 1 Internal Medicine, St. Joseph's Regional Medical Center, Paterson, USA; 2 Hematology and Oncology, St. Joseph's Regional Medical Center, Paterson, USA; 3 Internal Medicine, St. Joseph's Hospital Medical Center, Paterson, USA; 4 Internal Medicine, West Virginia School of Osteopathic Medicine, Lewisburg, USA; 5 Hematology and Oncology, St. Joseph's University Medical Center, Paterson, USA

**Keywords:** therapies, immunohistochemistry, heterologous tumor, non-small cell lung cancer (nsclc), pulmonary carcinosarcoma (pcs), pulmonary sarcomatoid carcinoma (psc)

## Abstract

Pulmonary carcinosarcoma (PCS) is a rare type of non-small cell cancer. Overall, middle-aged and older smokers are the most affected age and sex groups. The diagnosis of PCS is difficult due to the absence of characteristic imaging findings. Additionally, preoperative biopsies do not usually reflect the heterologous nature of this tumor. Given the rarity of such tumors and the challenging diagnosis, the prognostic factors have not been established, and the overall prognosis remains poor. The valid therapeutic options are still limited. Here, we report a rare case of metastatic PCS that was accidentally discovered by imaging and properly diagnosed after surgical resection. The clinicopathological features, diagnostic tools, genetic theories, prognosis, and therapeutic options of this rare cancer are also discussed.

## Introduction

Pulmonary sarcomatoid carcinoma (PSC) is one of the rare primary non-small cell lung cancers (NSCLCs). According to the World Health Organization (WHO) classification, PSC includes five different subtypes: pleomorphic carcinoma, spindle cell carcinoma, giant cell carcinoma, pulmonary blastoma, and carcinosarcoma [1,2]. Carcinosarcoma (CS) is characterized by a biphasic histopathological pattern consisting of malignant epithelial and sarcomatous mesenchymal components [3]. CS can arise in other body organs including the skin, uterus, esophagus, and hypopharynx [3]. Pulmonary carcinosarcoma (PCS) is a heterologous malignant tumor that can present as a symptomatic mass within the endobronchial tree or accidentally find mass at the peripheral lung parenchyma [4,5]. Accordingly, preoperative diagnosis is challenging. Biopsies usually show only one element of the tumor with subsequent misdiagnosis and delayed treatment of such highly aggressive rare tumors.

In this article, we present a 60-year-old male with a heavy smoking history who has accidental radiological findings of left lower lung mass. CT-guided biopsy failed to identify the sarcomatous element of the tumor. Status post left lower lobectomy and chest wall biopsy with a definitive diagnosis of metastatic PCS. Unusually, large cell neuroendocrine carcinoma was found to be the dominant carcinomatous component of the tumor. Histogenesis, clinical pictures, diagnosis, prognosis, and challenging treatment options are also discussed.

## Case presentation

A 62-year-old Hispanic male with a past medical history significant for anxiety, tobacco use disorder (25 pack-years), chronic obstructive lung disease, and pulmonary fibrosis presented to the emergency department (ED) with one week of shortness of breath. It was associated with a runny nose, cough, and anterior and posterior chest wall pain. He denied any fever, chills, cough, chest pain, palpitation, weight loss, or any other symptoms.

**Figure 1:**
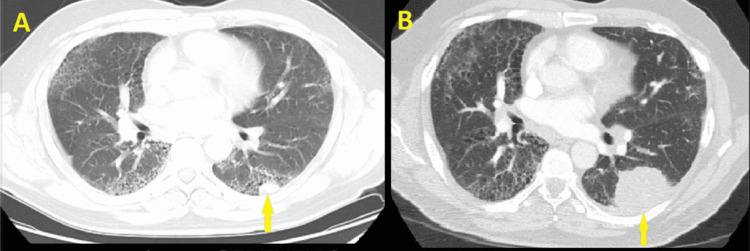
Computed tomography without contrast of the chest (A) and computed tomography angiography of the chest (B) (A) Computed tomography (CT) without contrast shows a mass-like area of consolidation seen toward the superior segment of the left lower lobe measuring 1.3 cm x 1.9 cm x 2.9 cm (yellow arrow). There is diffuse honeycomb changing in the bilateral lower lobes, right middle lobe, and peripherally along the upper lobes. There is subpleural consolidation in the upper lobes bilaterally to the apices, and no endobronchial lesion or central bronchiectasis. (B) CT angiography of the chest shows an interval growth of the previously reported left lower lobe nodule, presently measuring 7.1 cm x 3.9 cm, compatible with malignancy (yellow arrow). There is mediastinal and bilateral hilar lymphadenopathy, left greater than right. Positive emphysematous and fibrotic changes involve pulmonary parenchyma. There is no evidence of pneumothorax, central occlusive pulmonary embolism (PE), pulmonary arterial hypertension, right heart strain, pericardial effusion, or thoracic aortic dissection or aneurysm.

Nineteen months ago, the patient presented to the ED with concerning abdominal symptoms. Computed tomography (CT) abdomen was performed and accidentally relieved possible left lower lobe mass. Subsequently, a follow-up CT chest without contrast showed almost a mass-like area of consolidation in the superior segment of the left lower lobe measuring 1.3 cm x 1.9 cm x 2.9 cm, a pattern of pulmonary fibrosis, and bilateral hilar lymphadenopathy (Figure 1, Panel A). At that time, the patient decided to sign out against medical advice. Although he was instructed to follow up with his primary care doctor as an outpatient, he failed to seek further medical advice until he developed progressive worsening dyspnea that prompted this ER visit.

In the ED, vitals showed blood pressure of 121/63 mmHg, heart rate of 105 beats per minute, respiratory rate of 20 breaths per minute, and a temperature of 36.5 degrees Celsius. The patient’s oxygen saturation was 97% on room air. Physical exam was notable for mild wheezing and rales noted bilaterally as well as mild right paraspinal muscle tenderness. Laboratory studies were significant for an initial troponin level of 2 pg/mL with a brain natriuretic peptide (BNP) value of 14 pg/mL (Table 1). Electrocardiogram (EKG) is significant for sinus tachycardia, with no associated ST or T wave changes. Chest x-ray (CXR) was significant for diffuse interstitial opacities with mass-like density in the left midlung zone. Ultrasound venous lower extremities were negative for acute deep venous thrombosis. CT angiogram (CTA) of the chest was compatible with age-indeterminate left lower lobe pulmonary arterial embolus (PE). The CTA shows an interval growth of previously reported left lower lobe nodule/mass, presently measuring 7.1 cm x 3.9 cm. There was no evidence of central occlusive PE or right heart strain (Figure 1, Panel B). Oncology and pulmonology teams were consulted. Further imaging with CT head, abdomen, and pelvis was negative for metastatic disease.

**Table 1:** Initial laboratory values WBC: White blood count; RBC: Red blood count; Hgb: Hemoglobin; Hct: Hematocrit; MCV: Mean corpuscular volume; MCH: Mean corpuscular hemoglobin; MCHC: Mean corpuscular hemoglobin concentration; RDW: Red cell distribution width; PT: Prothrombin time; INR: International normalized ratio; PTT: Partial thromboplastin time; BUN: Blood urea nitrogen; ALP: Alkaline phosphatase; AST: Aspartate aminotransferase; ALT: Alanine aminotransferase; BNP: Brain natriuretic peptide; CK: Creatine kinase.

Test Name	Reading	Reference Range
Complete blood count		
WBC	9.6 K/mm^3^	4.5–11.0
RBC	4.64 K/mm^3^	4.33–5.83
Hgb	14.4 g/dL	13.5–17.5
Hct	42.0%	41–53
MCV	90.5 fL	80–100
MCH	31.0 pg	26–32
MCHC	34.3 g/dL	31–37
RDW	12.8%	0.5–16.5
Platelets	198 K/mm^3^	140–440
Neutrophils	70.2%	36.0–75.0
Lymphocytes	21.4%	24.0–44.0
Monocytes	5.0%	4.0–10.0
Eosinophils	2.0%	0.0–5.0
Basophils	0.4%	0.0–2.0
Routine coagulation		
PT	13.2 seconds	12.2–14.9
INR	1.0	N/A
PTT	33.5 seconds	21.3–35.1
D_Dimer Qnt	1.52 mcg/mL FEU	≤0.50
Routine chemistry		
Na	136 mEq/L	135–145
K	3.6 mEq/L	3.5–5.0
Cl	104 mEq/L	98–107
CO_2_	24 mEq/L	21–31
Glucose	136 mg/dL	70–110
Calcium	8.8 mg/dL	8.6–10.3
BUN	9 mg/dL	7–23
Creatinine	0.70 mg/dL	0.6–1.3
Total bilirubin	0.6 mg/dL	0.3–1.1
Total protein	6.8 g/dL	6.4–8.4
Albumin	4.5 g/dL	3.5–5.7
ALP	71 U/L	34–104
AST	21 U/L	13–39
ALT	15 U/L	7–52
Cardiac markers		
BNP	14 pg/mL	1–100
CK	78 U/L	30–223
Troponin-I	2 pg/mL	3–23

**Figure 2:**
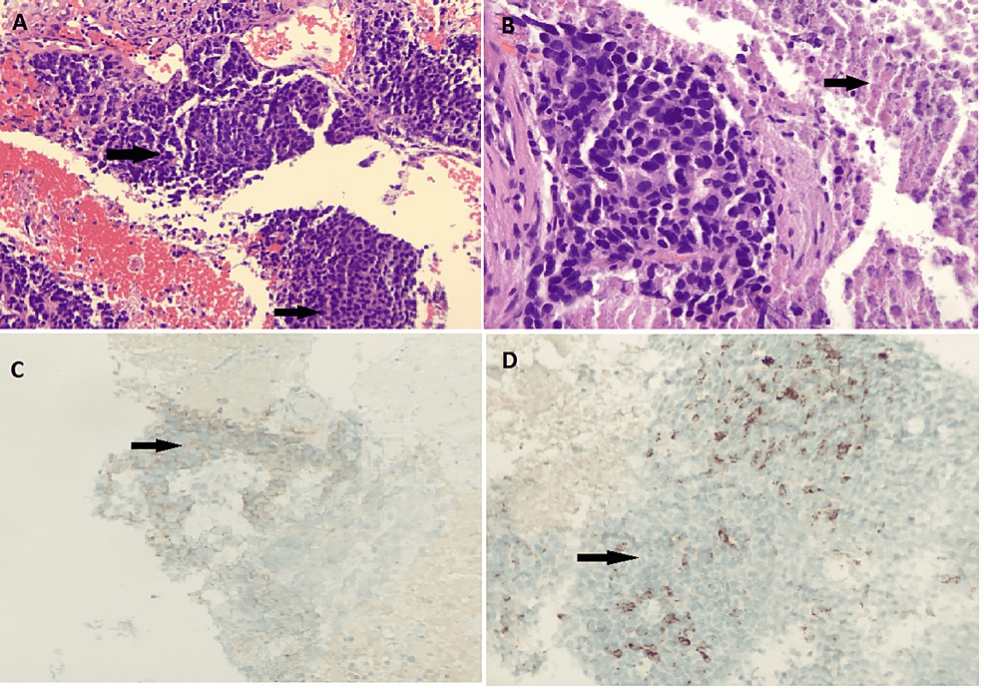
The CT-guided biopsy of the left lower lobe mass (A) The CT-guided biopsy of the left lower lobe mass shows atypical cells with eosinophilic cytoplasm indicating non-small cell carcinoma consistent with neuroendocrine carcinoma (arrow). (B) Brisk mitotic activity and focally extensive necrosis are present (arrow). Immunostains show the malignant neuroendocrine cells are focally and weakly positive for synaptophysin (arrow in C) and chromogranin (arrow in D).

The patient underwent a CT-guided biopsy of the left lower lung mass, which showed non-small cell carcinoma consistent with neuroendocrine carcinoma, extensively necrotic (Figure 2). Immunohistochemistry stains were strongly positive for CK7 and p53 and focally and weakly positive for synaptophysin and chromogranin; malignant cells are negative for CK20, thyroid transcription factor-1 (TTF-1), Napsin-1, p40, and CK5/6. A very high proliferation index (approximately 70%) is seen with Ki67 immunostaining.

The patient was discharged to follow up with oncology. Further MRI of the brain was negative for intracranial metastatic disease. Positron emission tomography (PET)-CT scan showed that multiple mediastinal lymph nodes are identified, ranging between 8 and 12 mm; however, they do not demonstrate significant fluorodeoxyglucose (FDG) uptake and are probably related to underlying fibrosis. The patient was referred to surgery. Status post left video-assisted thoracoscopic surgery (LVATS) that showed a suspicious area of tumor infiltration was appreciated in the posterolateral aspect of the chest wall, and a biopsy with an intraoperative frozen section was sent. Given the possibility of extensive metastatic disease, several samples for the intraoperative frozen section were obtained without evidence of metastatic disease. A decision was made for left thoracotomy with left lower lobectomy and mediastinal lymph node sampling (levels 10 R, 4L, 5, 6).

**Figure 3:**
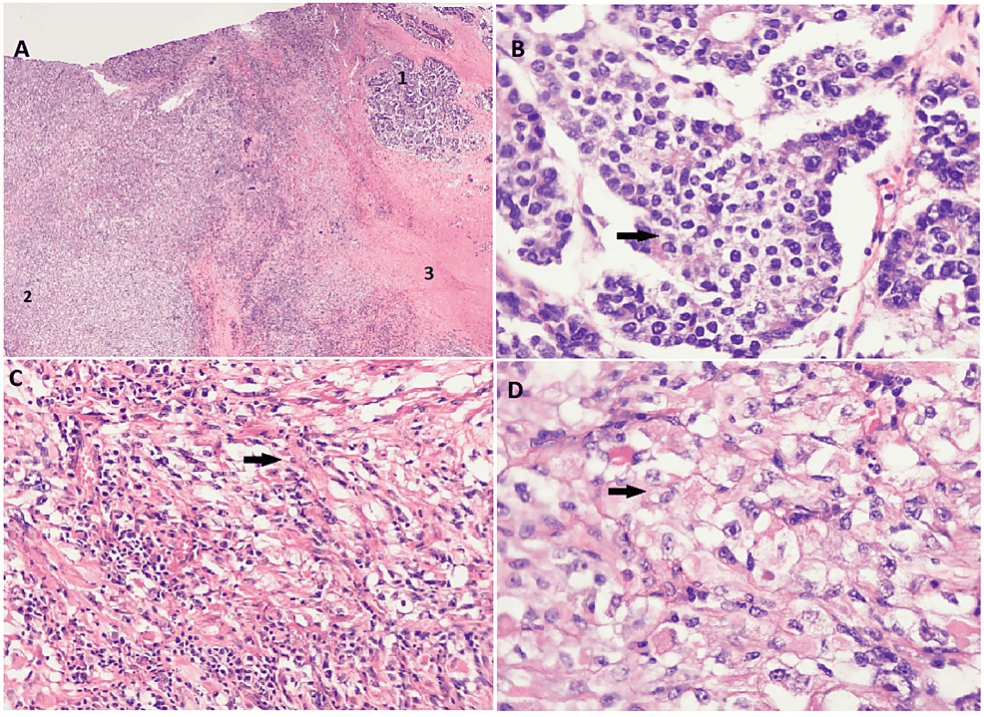
The postoperative histopathological imaging of the left lower pulmonary lobectomy The postoperative tissue (pulmonary lobectomy) is consistent with carcinosarcoma, which shows mixed components of multiple histological types with approximately 20% of large cell neuroendocrine carcinoma (Panels A1 and B) and 60% of sarcomatous components (Panels A2, C, and D). Focally, the carcinoma and sarcomatous components are intermixed. Extensive tumor necrosis is present (Panel A3). The sarcomatous component shows spindle cell morphology (Panel C), with areas of prominent rhabdoid differentiation (Panel D), suggestive of a heterologous element.

Pathology results showed PCS (mixed malignant neoplasm, including large cell neuroendocrine carcinoma 20%, small cell carcinoma 15%, spindle cell/sarcomatous component 60%) (Figure 3). The chest wall nodule showed malignant spindle cell neoplasm, consistent with metastatic carcinosarcoma of the lung primary. There was a focal lymphovascular invasion, but regional lymph nodes were negative for malignancy. Final pathology staging: pT4N0M1a consistent with prognostic stage IV. Given his good performance status, the oncology team proposed a tentative plan of molecular testing and pursuing palliative chemotherapy with carboplatin/paclitaxel for six cycles followed by RT evaluation. However, his condition has deteriorated and complicated with acute hypoxic respiratory failure secondary to hospital-acquired pneumonia and eventually family pursued comfort measures.

## Discussion

PCS accounts for <1% (0.2%-0.3%) of all primary pulmonary tumors, with a male to female ratio of 4:1. It usually presents in the seventh decade of life. Per recent literature, our patients correspond to the average age at diagnosis and range from 59 to 61 years [6]. The most important risk factor, as in our patient, is smoking which accounts for 90% of patients. Asbestosis was reported in a few cases [7,8].

Upon literature review, the carcinomatous component is mainly squamous cell carcinoma (69%), followed by adenocarcinoma (20%) and large cell carcinoma (11%). On the other hand, the most common mesenchymal component is poorly differentiated spindle cell sarcoma. However, lesions of rhabdomyosarcoma, osteosarcoma, and chondrosarcoma are often present [4]. Our patient represents subminorities of the population that was diagnosed with this classic primary PCS with large cell carcinoma being the dominant carcinomatous component.

The underlying histological origin of PCS is still under debate; however, there are three principal theories. The first theory suggests that the lesion arises from a multipotent cell that can differentiate into mesenchyme and epithelium. Another theory suggests that the tumor arises from epithelial or mesenchymal cell lines and subsequently undergoes metaplastic transformation [1,7]. Less likely is that PCS is an impingement tumor that arises from two separate epithelial and mesenchymal precursors [9,10].

In terms of clinical presentation, it has been shown that PCS has two subtypes [9]. Koss et al. have documented that the central endobronchial type is the most common and accounts for 62% of the cases [4]. It is usually slow-growing and accompanied by coughing, dyspnea, and hemoptysis [5]. The second less common type, as in our patient, is called peripheral invasive parenchymal carcinoma. This type is highly aggressive with early and widespread distant metastases. Unfortunately, patients with this subtype, like ours, are usually asymptomatic in the early stages. Thus, at the time of presentation, adjacent organs such as the mediastinum, pleura, or chest wall were usually involved [5,9].

CT radiographic findings in the literature usually show a solitary mass with an average tumor size of 7.0 cm, as in our patient. Contrary to the literature data, the radiographic findings of our patient showed a mass in the lower lobe instead of the recorded upper lobe [4,6]. Our patient's CT findings also show simultaneous obstructive pneumonia and changes in the pleura and parenchyma secondary to the mass effect. As per Qin et al., PCS can occur with extensive tumor necrosis, and subpleural lesions have a tendency to invade the chest wall or pleura, explaining the chest wall nodule identified in our patient [11]. Intratumoral ossification is rare, but calcification is common if osteosarcoma or chondrosarcomatous component was part of the tumor. Therefore, CT is used for only the primary identification of the tumor [6]. Only one study of 99 patients has reported that the mean positron emission tomography uptake is higher in sarcomatoid carcinoma than in other pulmonary tumors with P < 0.0001 [12]. In another study in 24 patients with PSC, a higher accumulation of 18F-FDG in PET/CT was noticed to be strongly associated with elevated PDL1 (programmed cell death receptor ligand-1) and KRAS (Kirsten rat sarcoma viral oncogene homolog) expression. Therefore, it determines the treatment regimen and prognosis of such cancer [13].

As regards the diagnosis, biopsies show the heterogeneous picture of the sarcomatous component as skeletal muscle or cartilage, or immunohistochemical stains for it can confirm the diagnosis [8]. However, given the rarity of the tumor, most biopsies fail to reflect the biphasic nature and subsequently misdiagnose or even delay the treatment. Therefore, the gold standard for diagnosing this rare cancer is surgical removal and immunohistochemical staining [6].

In terms of the prognosis and overall survival (OS) of this aggressive tumor, the recent literature review of multiple studies reported the median survival of around 21 to 22 months with five-year survival rates ranging from 11% to 25%, which is inferior compared to other NSCLCs [6]. It was blamed on its frequent genetic mutation and resistance to first-line chemotherapy [14,15]. Within the scope of this view, a question came to our mind related to our patient's case. Would our patient's prognosis or OS be different if he sought medical advice and treatment earlier (while the tumor was smaller)? Surprisingly, all studies concluded that there is no clinically significant correlation between OS and age, smoking, tumor size, pleural, or lymphatic metastasis [6]. Besides, it was found that pN0 does not affect the prognosis as more than half of the patients without lymphatic metastasis have vascular invasion and are thus considered distant metastasis [6]. Additionally, Ung et al. reported that adjuvant chemotherapy would not make a potential significance on the prognosis [16]. In contrast, according to the data, if our patient presented with pathological lower stages, he would have a higher survival probability [17]. Furthermore, data showed that time from symptom onset to diagnosis, the extent of surgical resection, tumor growth, and tumor invasiveness represent independent prognostic factors and would make a difference in our patient prognosis [6].

In terms of treatment options for this highly aggressive tumor, surgical removal is the main corner of treatment if possible [6,18]. According to evidence-based medicine, PCS is currently managed with standard treatments used for NSCLC [15,18,19]. The standard treatments include complete early surgical resection combined with adjuvant therapy (with platinum-based combination chemotherapy and radiotherapy), which may increase the survival of lung cancer patients as per Maneenil et al.'s trial [6,16,20]. Although our patient has an advanced stage, his complete tumor resection and the identified chest wall nodule would extend his survival period if he received adjuvant therapy. Unfortunately, he died from a non-cancer-related disease (pneumonia) after surgery, which excludes him from the treatment-failure or -resistant group [20,21]. Furthermore, given the rarity of this disease, there are no randomized control trials on the long-term treatment or follow-up surveillance. Nowadays, researchers are trying to involve victims of this highly aggressive tumor in new treatments with biologically targeted therapy than the traditional therapy such as anti-estimated glomerular filtration rate (anti-EGFR) drugs, anaplastic lymphoma kinase (ALK) gene expression inhibitors, KRAS gene mutation inhibitors such as CDK 4/6 inhibitors or mitogen-activated protein kinase (MEK) inhibitors, and immune-checkpoint inhibitors (ICIs) [16,22,23]. Recent trials reported that these new biological target therapy could be used in patients with drug resistance after radiotherapy and chemotherapy [23].

## Conclusions

PCS is a rare highly malignant primary pulmonary tumor. Contrary to previous cases, this tumor can also affect the lower lung lobes. A preoperative diagnosis of PCS is challenging because of the heterologous nature of the tumor structures. This case report aims to increase the physician’s awareness of such rare yet highly malignant and rapidly advanced tumors. Emphasize the gold standards to confirm its diagnosis and the influence of time to diagnosis on the prognosis. Complete surgical resection is still the only effective treatment for PCS, but the prognosis remains very poor. Clinical and prognostic factors of PCS are still debatable. Given the rarity of this tumor, further studies and case series are needed to identify factors that influence survival outcomes in order to optimize the clinical practice guidelines.
